# Clinico Microbiological Profile of Diabetic Foot Ulcers: A Retrospective Study

**DOI:** 10.7759/cureus.95291

**Published:** 2025-10-24

**Authors:** Liza Das, Shradha Smriti, Rajesh K Dash, Nipa Singh, Subhra Snigdha Panda, Ipsa Mohapatra, Niranjan Moharana, Bansidhar Mulia, Sambit Das, Dipti Pattnaik

**Affiliations:** 1 Department of Microbiology, Kalinga Institute of Medical Sciences, Bhubaneswar, IND; 2 Department of Community Medicine, Kalinga Institute of Medical Sciences, Bhubaneswar, IND; 3 Department of General Surgery, Kalinga Institute of Medical Sciences, Bhubaneswar, IND; 4 Department of Plastic Surgery, Kalinga Institute of Medical Sciences, Bhubaneswar, IND; 5 Department of Endocrinology, Kalinga Institute of Medical Sciences, Bhubaneswar, IND

**Keywords:** antibiotic susceptibility, diabetes mellitus, diabetic foot ulcers, gram negative, gram positive

## Abstract

Introduction and aim: Diabetic foot ulcer (DFU) is a frequent and critical complication associated with diabetes mellitus. They carry a higher risk of infection, longer hospital stays, and increased healthcare costs. This study aimed to identify the microbiological profile and antibiotic susceptibility pattern of organisms isolated from infected diabetic foot ulcers.

Material and methods: A retrospective, record-based, cross-sectional study was conducted by retrieving data on culture isolates obtained from diabetic foot ulcers and the clinical data of these patients over a period of two years (from August 2022 to August 2024) at a tertiary care hospital. The demographic, clinical, and laboratory data were retrieved from the laboratory information system; all data were entered into an Excel spreadsheet (Redmond, WA: Microsoft Corp.), and the variables were analyzed.

Results: A total of 100 patients with DFUs, and the details of 156 samples obtained from the diabetic foot ulcers of these patients, were included in the study. Among the 100 patients, the majority were male (76%, 76/100), and the age group most commonly affected was 50-60 years (40%, 40/100). Pus was the predominant sample (41.7%, 65/156) among the total 156 samples collected. Among the 156 samples collected, 62.2% (97/156) showed mono-microbial growth, and 37.8% (59/156) showed poly-microbial growth. The total number of bacterial and fungal isolates was 213 and five, respectively. All fungal isolates were identified to be *Candida tropicalis*. The most common bacterial isolates were *Pseudomonas aeruginosa* (18.8%, 40/213), *Klebsiella pneumoniae* (17.8%, 38/213), *Escherichia coli* (14.1%, 30/213), and Enterococcus spp. (12.7%, 27/213), and *Staphylococcus aureus *(11.7%, 25/213). The most effective antibiotics for *Pseudomonas aeruginosa *were carbapenems, imipenem (90%, 36/40) and meropenem (80%, 32/40), followed by piperacillin-tazobactam (75%, 30/40) and amikacin (75%, 30/40). Imipenem (55%, 21/38) was the most sensitive antibiotic for *Klebsiella pneumoniae* followed by gentamicin (45%, 17/38). For Gram-positive isolates, Enterococcus spp. was most sensitive to teicoplanin and linezolid (100%, 27/27), followed by ampicillin (96.3%, 26/27) and vancomycin (77.8%, 21/27). *Staphylococcus aureus *isolates were most sensitive to vancomycin and teicoplanin (92%, 23/25), followed by daptomycin (84%, 21/25). A total of 25 *Staphylococcus* *aureus* isolates were obtained, of which 92% (23/25) were found to be methicillin-resistant *Staphylococcus aureus* (MRSA).

Conclusion: This study has enhanced our understanding of the epidemiology of diabetic foot ulcers by identifying common pathogens. The antibiotic susceptibility pattern of the pathogens will help clinicians provide better empirical therapy, thereby promoting faster wound healing.

## Introduction

Diabetes mellitus (DM) affects about 422 million people worldwide and causes two million fatalities each year. One of the most serious outcomes of poorly managed and long-lasting diabetes is the development of diabetic foot ulcers (DFUs), which typically manifest as ulcerations at the bottom of the foot [1].

DFUs are among the most severe complications of diabetes mellitus, and the development of foot ulcers in patients with diabetes has been estimated to be between 19% and 34% throughout their lifetime. DFU is a leading cause of hospital admission, increased morbidity, and limb amputations [2]. Infected DFUs, also referred to as diabetic foot infections (DFIs), present significant morbidity and increase the risk of mortality [3].

DFUs refer to lesions in the skin epidermis that penetrate deeper and disrupt the dermal layer. DFUs develop due to a combination of diabetic sensory, motor, and autonomic neuropathy. They typically appear on areas of the foot subjected to repeated pressure and trauma [4]. It frequently results in lower limb amputation and osteomyelitis [5].

The bacterial profile of DFI changes with the severity of the condition. Common pathogens isolated from DFIs include aerobic Gram-positive bacteria, such as *Staphylococcus aureus*, *Streptococcus pyogenes,* and Enterococcus spp., and Gram-negative bacteria, such as *Escherichia coli*, *Klebsiella pneumoniae*, *Pseudomonas* *aeruginosa*, Acinetobacter spp., and Proteus spp. [6].

Patients with diabetes are particularly at risk of infections, which often result in the frequent use of broad-spectrum antibiotics and the development of antimicrobial resistance (AMR) [7]. Treatment of DFUs has become difficult because of poly-microbial infections and the rise in antibiotic resistance [8]. Multidrug-resistant pathogens infecting DFUs lead to slower wound healing, longer hospitalizations, increased risk of amputation, higher healthcare costs, and limited treatment options [9-11]. It is necessary to identify the pathogens associated with DFIs and to know the local antibiogram to guide clinicians in choosing empirical treatment and preventing inappropriate antibiotic use. Proper treatment of DFUs will prevent antimicrobial resistance, which has been emphasized by the World Health Organization (WHO) as a serious public health challenge [12]. This study aimed to determine the microbiological profile and antibiotic susceptibility pattern of organisms isolated from infected foot ulcers of patients with diabetes.

## Materials and methods

Study design and setting

This is a retrospective, record-based, cross-sectional study extending for a period of two years (from August 2022 to August 2024). The clinical and microbiological data of patients with diabetic foot ulcers (admitted to the departments of general surgery, plastic surgery, and endocrinology at a tertiary care hospital), whose samples were sent to the microbiology laboratory for culture, were retrieved from the laboratory information system (LIS).

Inclusion and exclusion criteria

**Figure 1:**
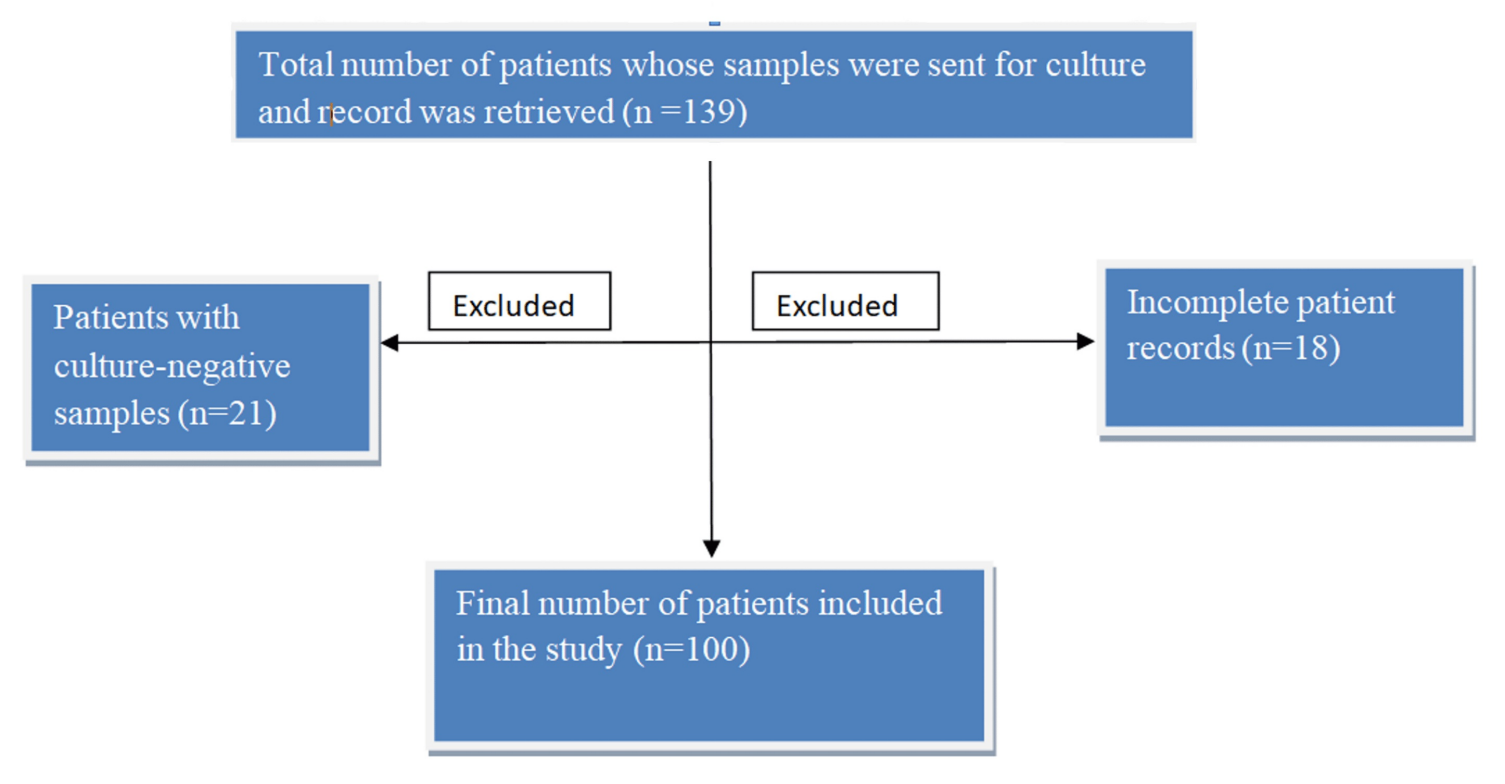
Flowchart representing the total number of patients with diabetic foot ulcers included in the study (n=100).

The data of inpatient samples aged >18 years diagnosed with DFU, with a culture-positive sample from the ulcer, were included. Data from outpatients, duplicate samples from the same patient, culture-negative samples, and samples with unavailable culture reports were excluded. A flowchart illustrating the selection process for patient data with DFU is shown (Figure 1).

Of the 139 patient samples received in the laboratory for culture, data from 39 patients were excluded due to either a negative culture report or the unavailability of complete patient information. This study included the demographic, clinical, and microbiological data of 100 patients.

Sample collection

As a routine practice, the samples from patients with DFUs are usually collected on the first day of admission to the hospital in different departments (surgery, plastic surgery, and endocrinology). Pus samples are aspirated from the deep portion of the ulcer base using a sterile syringe, after cleaning the wound surface with sterile saline. The aspirates are then transferred into a sterile container and sent to the laboratory for culture. First, the overlying debris was removed with a scalpel, and then deep tissue samples were collected from the areas within and adjacent to the infected sites under aseptic conditions. Then, the tissue sample was placed inside a sterile container and transported to the laboratory for culture. The swab samples are obtained by gently rotating sterile swabs on the base of the ulcer [13]. As per the clinicians’ instructions, samples were collected from different sites, and in some cases, more than one sample type was sent to the microbiological laboratory.

Microbiological processing of the samples

Specimens, such as pus, deep tissue, and wound swabs, were collected and inoculated on blood agar, MacConkey agar, and Sabouraud Dextrose agar (SDA) plates. The blood agar and MacConkey agar plates were incubated at 37°C for 24 hours. The plates showing growth of more than one bacterial isolate were again subcultured on MacConkey agar plates to obtain pure isolated colonies. The SDA plates were incubated until fungal growth was observed or for a maximum of seven days. If no growth was seen after seven days, the result was reported as negative for fungal growth [14].

The identification and antibiotic sensitivity testing of the isolates were done by the automated VITEK 2 compact system (Marcy‑l’Étoile, France: BioMérieux). The results were interpreted according to the Clinical and Laboratory Standards Institute (CLSI) 2024 guidelines [15]. In case multiple samples (>1) were obtained from a single patient, the pathogens isolated from the specimens were screened, and if it was noted that the isolates were similar, only a single isolate was included in the study to avoid duplication. Multidrug-resistant (MDR) bacteria were defined as “acquired non-susceptibility to at least one agent in three or more antimicrobial categories” [16]. Extended-spectrum β-lactamases (ESBLs) are a group of enzymes that confer resistance to penicillins, as well as first-, second-, and third-generation cephalosporins, and aztreonam (but not cephamycins and carbapenems), thereby making these antibiotics ineffective [17]. Organisms that show resistance to any one of the carbapenem antibiotics (imipenem, meropenem, ertapenem, or doripenem) are referred to as carbapenem-resistant organisms [18].

Methicillin-resistant *Staphylococcus aureus *(MRSA) is a strain of *Staphylococcus aureus* that is resistant to a broad class of antibiotics known as β-lactams, which includes penicillins, cephalosporins, and carbapenems [19].

Statistical analysis

The data were recorded in an Excel spreadsheet (Redmond, WA: Microsoft Corp.), and analysis was conducted using SPSS version 27 (Armonk, NY: IBM Corp.). Continuous data were expressed as mean±SD and interquartile range. Quantitative discrete data were expressed as frequency in numbers, percentages, and proportions.

## Results

A total of 100 patients were included in the study. The mean age of patients was 55.4±10.7 (range: 34-85) years, with a male:female ratio of 3.2:1; 76% were males (76/100) and 24% were females (24/100). The average duration of diabetes mellitus was 11.1±6.5 years (minimum of 4 and maximum of 25 years). The mean glycated hemoglobin (HbA1c <9) level was 4.5±2.21, and 58% (58/100) (HbA1c >9) had a mean of 9.4±5.2. The mean albumin level was 3.15±0.64 g/dL, and the mean creatinine level was 1.07±0.51 mg/dL. The baseline demographic and clinical characteristics of all the patients are shown in Table 1.

**Table 1:** Baseline demographic and clinical characteristics of patients with diabetic foot infections (n=100). DFU: diabetic foot ulcer

Demographic characteristics and clinical parameters of patients with DFU	Findings
Age (mean±SD in years)	55.4±10.7
Male:female	3.2:1
Duration of diabetes mellitus (mean±SD in years)	11.1±6.5
Duration of diabetic foot ulcer	
>60 days, n (%)	67 (67%)
<60 days, n (%)	33 (33%)
Hypertension, n (%)	44 (44%)
HbA1c >9 (%, mean±SD)	9.4±5.2
Insulin use, n (%)	37 (37%)
Albumin (g/dL, mean±SD)	3.15±0.64
Serum creatinine (mg/dL, mean±SD)	1.07±0.51
Haemoglobin (g/dL, mean±SD)	109.4±25.2
Duration of hospitalization (days, mean±SD)	25.1±19.1
Amputation, n (%)	41 (41%)

**Figure 2:**
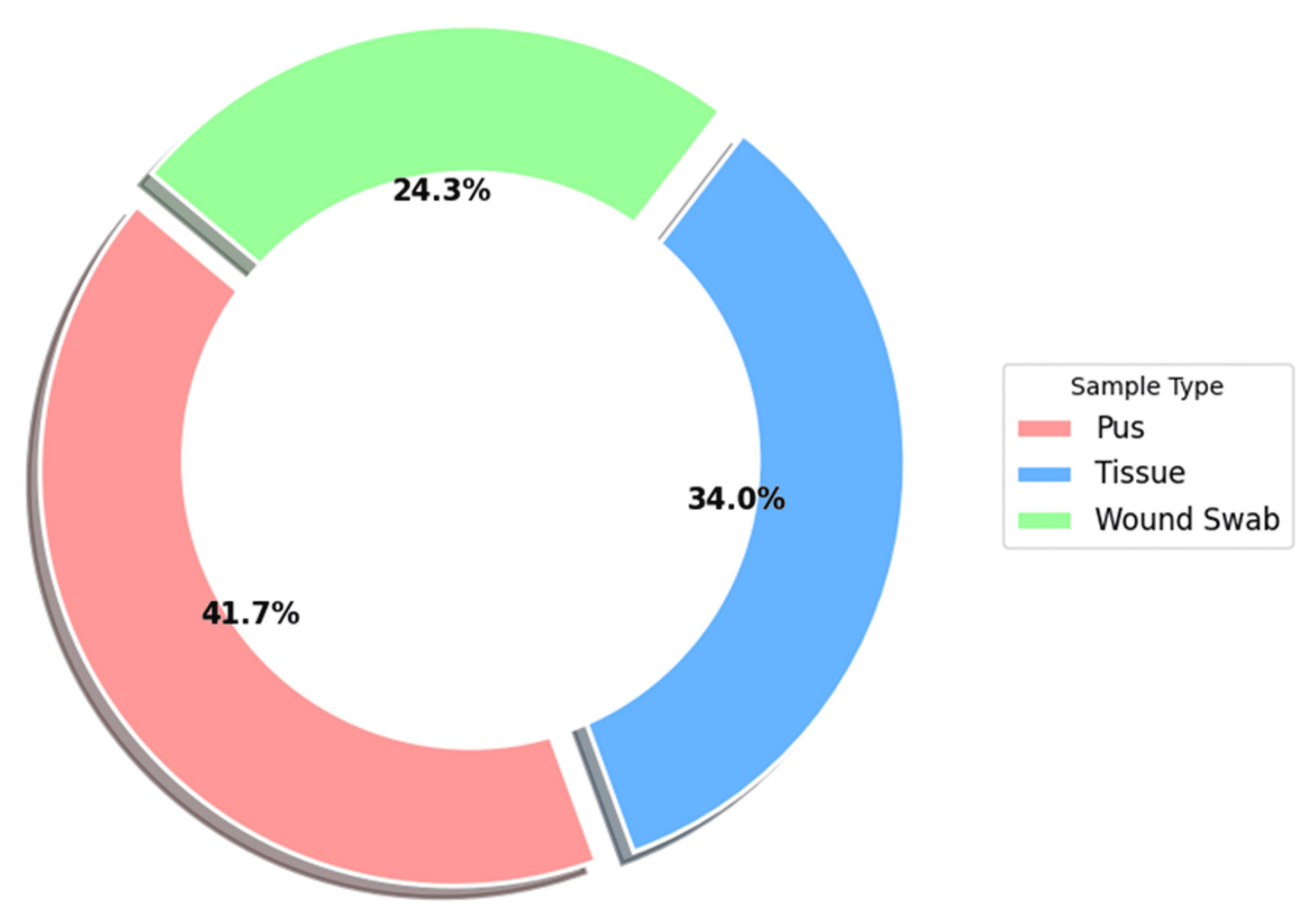
Specimen-wise distribution (in percentage) of clinical isolates obtained from DFU (n=156). DFU: diabetic foot ulcer

A total of 156 specimens were obtained from the diabetic foot ulcers of 100 patients, of which pus comprised the majority (41.7%, 65/156), followed by deep tissue and wound swab samples, being 34% (53/156) and 24.3% (38/156), respectively (Figure 2).

Among the 156 specimens, mono-microbial growth was observed in 62.2% (97/156) specimens, whereas poly-microbial growth was observed in 37.8% (59/156). A total of 218 culture isolates were obtained, of which 213 were bacterial and five were fungal isolates. A total of 74.6% (159/213) were Gram-negative isolates and 25.4% (54/213) were Gram-positive isolates.

**Figure 3:**
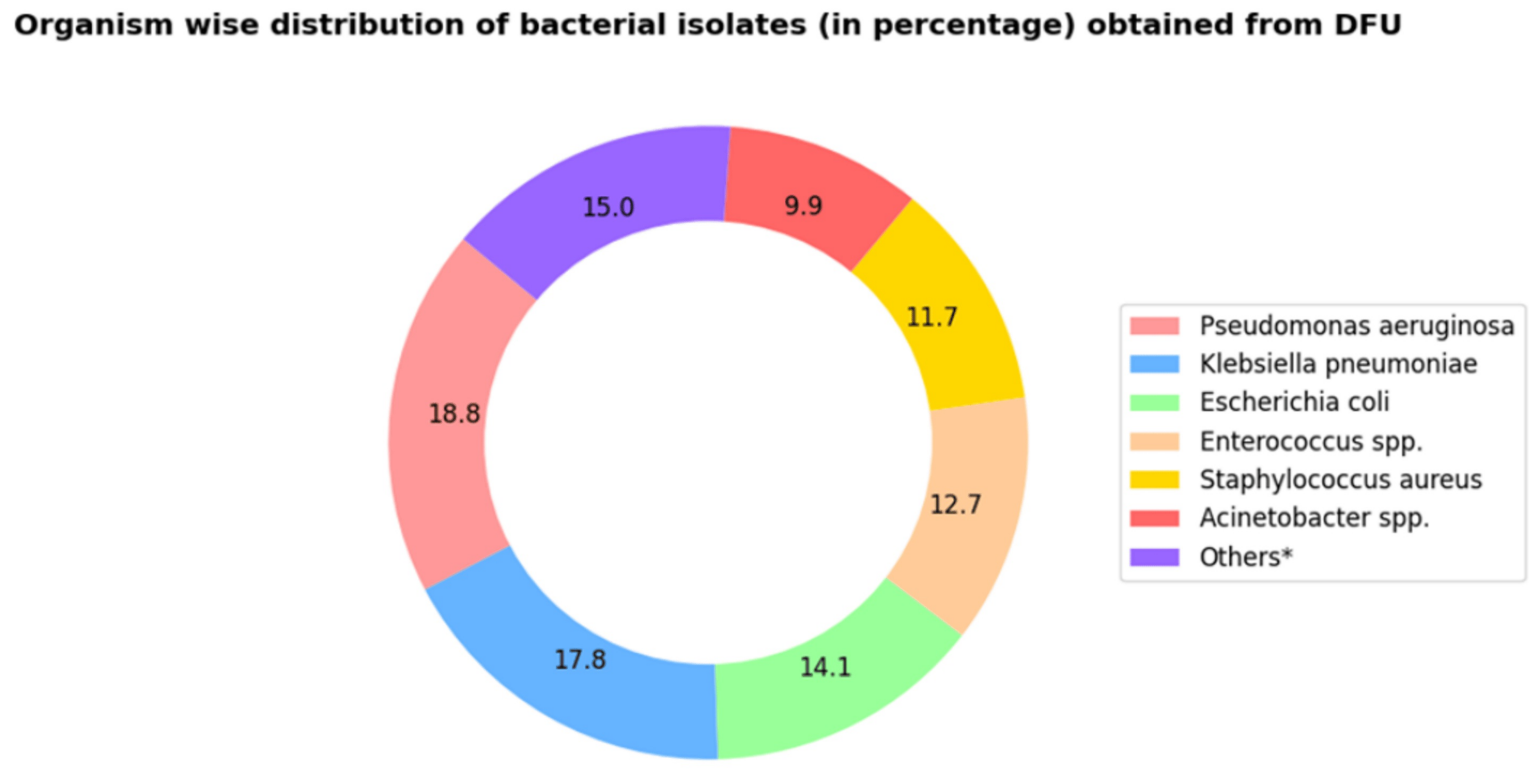
Distribution of bacterial pathogens isolated from clinical samples of diabetic foot ulcers illustrating both Gram-negative and Gram-positive bacteria (n=213). *Others denotes *Proteus mirabilis*, *Enterobacter aerogenes*, *Morganella morganii*, *Citrobacter freundii*, *Serratia marcescens*, *Sphingomonas paucimobilis,* and *Streptococcus anginosus*, which together constitute 15% (n=213).

Among the Gram-negative bacteria (GNB) isolated, *Pseudomonas* *aeruginosa* comprised 18.8% (40/213), followed by *Klebsiella* *pneumoniae* (17.8%, 38/213), *Escherichia* *coli* (14.1%, 30/213), and Acinetobacter spp. (9.9%, 21/213). The less frequently isolated GNB were *Proteus* *mirabilis* (4.7%, 10/213), *Enterobacter* *aerogenes* (2.8%, 6/213), *Morganella* *morganii* (1.9%, 4/213), *Citrobacter* *freundii* (1.9%, 4/213), *Serratia* *marcescens* (1.4%, 3/213), and *Sphingomonas paucimobilis* (1.4%, 3/213). Among the Gram-positive bacteria isolated, the majority of the isolates were Enterococcus spp. (12.7%, 27/213) followed by *Staphylococcus* *aureus* (11.7%, 25/213) and *Streptococcus* *anginosus* (0.9%, 2/213) (Figure 3).

The most common Gram-negative bacteria were *Pseudomonas* *aeruginosa* (18.8%, 40/213) and *Klebsiellapneumoniae* (17.8%, 38/213). Enterococcus spp. (12.7%, 27/213) and *Staphylococcus* *aureus* (11.7%, 25/213) were the common Gram-positive bacteria isolated from DFU. Five fungal isolates were also obtained, all of which were identified as *Candida* *tropicalis*. The antimicrobial susceptibility test showed that the fungal isolates were susceptible to all the tested antifungal agents (amphotericin B, caspofungin, flucytosine, fluconazole, micafungin, and voriconazole).

**Figure 4:**
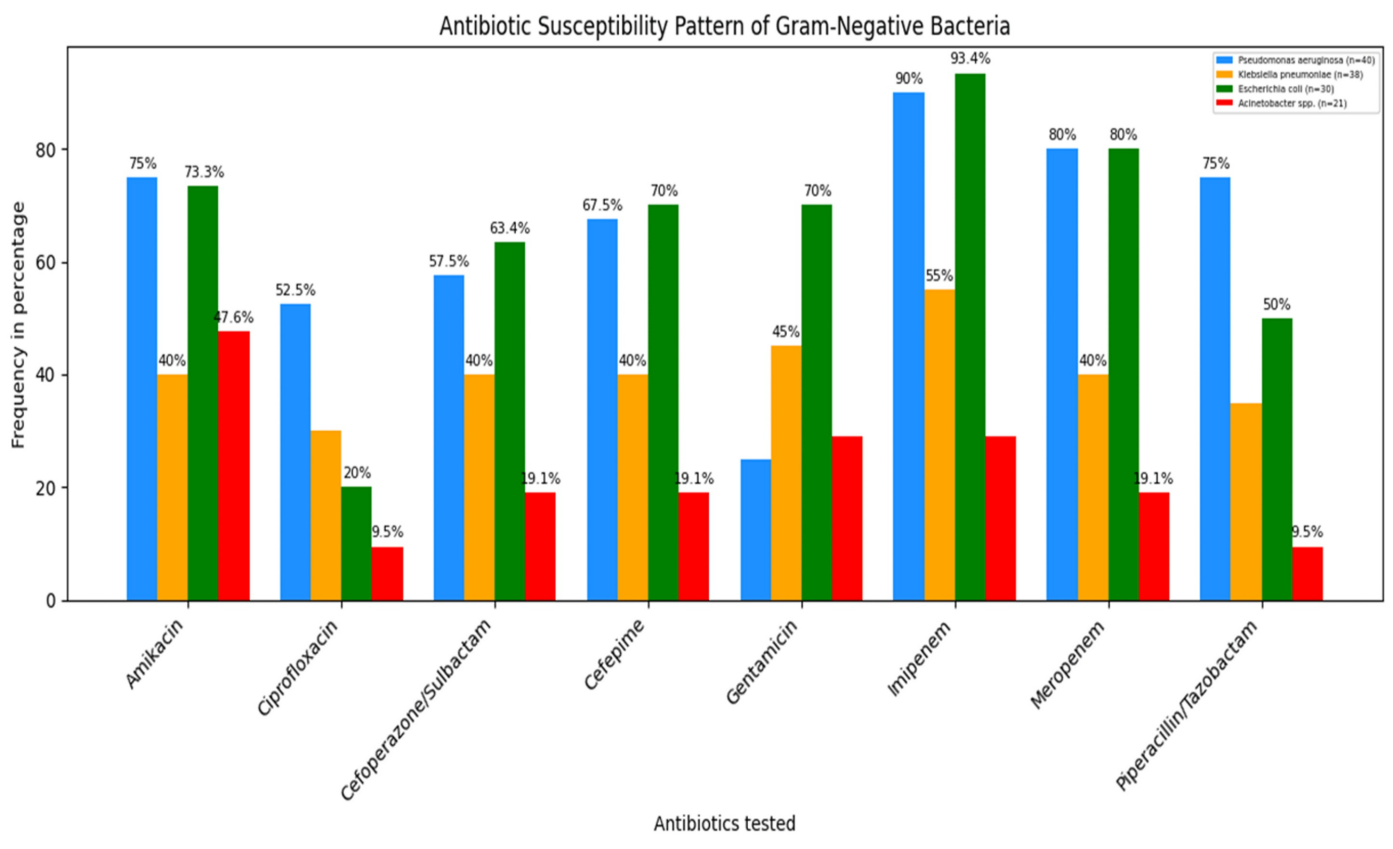
Antibiotic susceptibility pattern of commonly isolated Gram-negative bacteria from DFU (n=159). DFU: diabetic foot ulcers

Among the 159 Gram-negative isolates, there were 40 *Pseudomonas* *aeruginosa* and 38 *Klebsiella* *pneumoniae* isolates. *Pseudomonas* *aeruginosa* isolates were most sensitive to carbapenems (imipenem 90%, 36/40 and meropenem 80%, 32/40), followed by piperacillin-tazobactam (75%, 30/40) and amikacin (75%, 30/40). Imipenem (55%, 21/38) was the most sensitive antibiotic for *Klebsiella* *pneumoniae,* followed by gentamicin (45%, 17/38) (Figure 4).

*Pseudomonas* *aeruginosa*, *Klebsiella* *pneumoniae,* and *Escherichia* *coli* showed the most significant susceptibility to imipenem, and Acinetobacter spp. was most susceptible to amikacin.

Resistance markers among Gram-negative isolates

ESBL producers - of the total 159 Gram-negative bacterial (GNB) isolates, *E. coli*, *K. pneumoniae*, and *Proteus mirabilis *together constituted 78 isolates, of which 50% (39/78) were identified as ESBL producers according to the CLSI 2024 guidelines. CRE - the total number of Gram-negative bacterial isolates was 159, of which 39% (62/159) were identified as CRE according to the CLSI 2024 guidelines. MDR among Gram-negative isolates - among the 159 Gram-negative isolates, 31% (50/159) were MDR, with *K. pneumoniae *accounting for 58% (29/50) of the MDR strains, making it the predominant MDR species.

**Figure 5:**
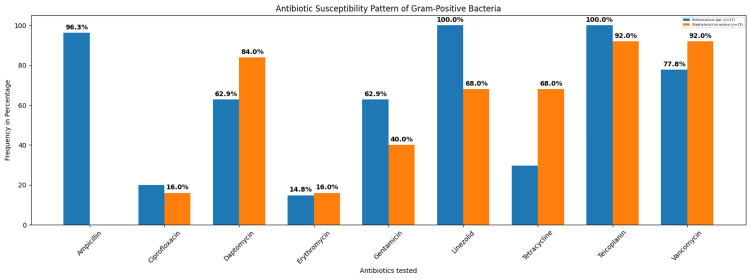
Antibiotic susceptibility pattern of commonly isolated Gram-positive bacteria from DFU (n=54). DFU: diabetic foot ulcers

Among the 54 Gram-positive isolates, Enterococcus spp. accounted for 27 isolates, and *Staphylococcus* *aureus* for 25 isolates. Enterococcus spp. was most sensitive to both teicoplanin and linezolid (100%, 27/27), followed by ampicillin (96.3%, 26/27) and vancomycin (77.8%, 21/27). *Staphylococcus* *aureus* was most sensitive to both vancomycin (92%, 23/ 25) and teicoplanin (92%, 23/25), followed by daptomycin (84%, 21/25) (Figure 5). A total of 25* Staphylococcus aureus* isolates were obtained, of which 92% (23/25) were found to be methicillin-resistant *Staphylococcus aureus *(MRSA). Enterococcus spp. and *Staphylococcus* *aureus* showed maximum susceptibility to teicoplanin.

Resistance markers among Gram-positive isolates

MRSA - a total of 25 *Staphylococcus aureus *isolates were obtained, of which 92% (23/25) were found to be MRSA, as per the CLSI 2024 guidelines.

## Discussion

Diabetic foot ulcers (DFUs) are a serious complication of diabetes mellitus, and if left untreated, they can lead to bacterial infections, gas gangrene, osteomyelitis, or amputation. Bacterial infections of DFUs may delay wound healing, prolong recovery time, increase treatment costs, and increase amputation risks [20]. In India, the number of diabetic foot ulcer patients is rising across both urban and rural areas [21]. Proper diabetes-related foot care remains the least prioritized practice in India [22]. Factors such as barefoot walking, poor hygiene, inadequate diabetes control, lack of education among people, and delayed presentation of foot ulcers aggravate foot injuries, leading to infections [23].

In the present study, the age of patients ranged from 35 to 81 years, and the mean age of patients with DFU was 55.4±10.7 years. A review from India reported that various studies have found the mean age of patients with DFU to be 56.39 years, consistent with our findings [24]. Further, Khan et al. observed that the DFU was more common within the age group of 56-65 years (38.33%), while the age group 25-35 years was less affected (10%) [25]. Therefore, these findings suggest that individuals within the age range of 56-65 years are more likely to be affected by DFUs. The risk of developing DFU increases with age, likely due to prolonged diabetes, persistent uncontrolled hyperglycemia, and associated comorbidities [4].

The male:female ratio with DFU was 3.2:1, indicating that males are at a higher risk of ulcer development than females. Similar findings have also been reported in other studies, indicating that males are predominantly affected by DFU, which might be due to certain occupational activities in men that increase the risk of foot trauma and subsequent ulceration [26].

We found that the mean duration of diabetes among the patients was 11.1±6.5 years, which is slightly higher than that reported by Kumar et al. (6.38±4.57 years), Boopesh et al. (8.13±4.28 years) [27,28]. A Chinese study observed the mean duration of diabetes in patients with DFU to be 13.1±7.5 years, which is consistent with our findings [29]. Persistent hyperglycemia increases the likelihood of developing a diabetic foot ulcer by promoting oxidative stress and impairing cellular function, leading to delayed wound healing [30]. The period for which the patients were affected by diabetic foot ulcer prior to hospitalization was more than 60 days in 67% (n=100) of patients, which is considerably higher compared to the findings of Smith-Strøm et al. (38.1%) [31]. Another study indicated that the severity of DFU is influenced by factors such as a longer duration of diabetes mellitus, prolonged foot ulcer duration, and infected ulcers [32].

Hypertension, along with diabetes mellitus, is an important risk factor for the development of DFU [33]. In this study, 44% (44/100) of the patients had hypertension. However, several studies have reported that hypertension is more frequent among patients with DFU (70-90%) [34,35].

In this study, the mean HbA1c level was 9.4%±5.2, indicating poor glycemic control in majority of the patients. Chronic hyperglycemia is a well-established risk factor for infections and impaired wound healing in patients with diabetes. Similar to our findings, Jawzali et al. observed a mean HbA1c of 9.04% ±1.7 in patients with DFU [36]. Various studies have indicated that elevated levels of HbA1c (>6.5%) are strongly associated with delayed wound healing, resulting in longer duration of ulcer and recurrence [37,38]. Approximately 37% (n=100) of the patients with DFU were on insulin therapy. Kasturi et al. observed that patients taking insulin are at a higher risk of development of DFU [39]. A considerable proportion of DFU patients in our study required amputation (41%, 41/100). This is comparable to the findings of the study by Shabhay et al. from northeastern Tanzania, who reported an amputation rate of 51.67% [40].

We observed that maximum bacterial isolates (41.7%, 65/156) were isolated from the pus samples, whereas several other studies have reported deep tissue as the predominant sample type, followed by pus [24,41]. Of all the samples yielding positive cultures, mono-microbial growth was observed in 62.2% (97/156) of the samples, while 37.8% (59/156) exhibited poly-microbial growth. Our observations correlate with the findings of Macdonald et al., indicating that mono-microbial infections are more common in diabetic foot ulcers [3].

In this study, *Pseudomonas* *aeruginosa* was the predominant Gram-negative bacteria isolated (18.8%, 40/213), followed by *Klebsiella pneumoniae *(17.8%, 38/213) and *Escherichia* *coli* (14.1%, 30/213). Various studies have reported *Pseudomonas aeruginosa *to be the most prevalent isolate obtained from DFU [29,42]. However, in contrast to our findings, an Indian study observed that *K*. *pneumoniae* was the most frequently isolated organism [8]. Among the Gram-positive bacteria, Enterococcus spp. was frequently isolated (12.7%, 27/213), followed by *Staphylococcus* *aureus* (11.7%, 25/213). Many other studies have reported that *S*. *aureus* and Enterococcus spp. are the most prevalent microorganisms isolated from DFU [3,43].

*P*. *aeruginosa* demonstrated the highest sensitivity to carbapenems (especially imipenem and meropenem), while amikacin and piperacillin-tazobactam both showed 75% susceptibility. These results are comparable with previous studies reporting high effectiveness of carbapenems, amikacin, and piperacillin-tazobactam against *P*. *aeruginosa* [8,44]. In the present study, *K. pneumoniae *showed high susceptibility to imipenem (55%), which is in agreement with observations documented in previous reports [45,46]. However, Govindaswamy et al. have observed that *K*. *pneumoniae* was highly sensitive to tigecycline and ertapenem (94%), followed by imipenem (88%) [41].

Enterococcus spp. demonstrated high susceptibility to linezolid and teicoplanin (100%), followed by ampicillin (96.3%) and vancomycin (77.8%). These findings are partially aligned with those of Jain and Barman and Coşkun et al., who reported 100% susceptibility to linezolid but varied sensitivity towards teicoplanin (80-90%). Another study done by Boccella et al. reported 98.1% susceptibility to ampicillin [43,47,48].

Vancomycin and teicoplanin (92%) were highly effective against *Staphylococcus* *aureus*, followed by daptomycin (84%). Similar observations have been reported in previous studies, where vancomycin consistently showed higher sensitivity against *S*. *aureus* (90-100%) [43,44]. However, a study by Atlaw et al. documented lower sensitivity rates of vancomycin and daptomycin (60-70%), which is in contrast to our findings [20].

Screening for resistant biomarkers in bacterial isolates from DFU revealed that 31% (50/159) of the GNB exhibited multidrug resistance (MDR), with *K*. *pneumoniae* being the most prevalent (58%, 29/50). Costa et al. reported that MDR *K*. *pneumoniae* accounted for approximately 16% of cases of diabetic foot infection [49]. In our study, 50% (39/78) of the GNB were ESBL-producers, and 39% (62/159) were carbapenem-resistant Enterobacterales (CRE). Among Gram-positive bacteria, methicillin-resistant *S*. *aureus *(MRSA) accounted for 92% (23/25). These findings are comparable with a study that reported ESBL-producing GNB to be 53.9%, 16.2% to be CRE, and 81.3% to be MRSA obtained from DFIs [50]. Similarly, an Indian study by Madhukar et al. documented 16.2% ESBL-producing GNB and 40% MRSA, findings that partially align with our observations [51].

This study highlights that both Gram-positive and Gram-negative bacteria are major contributors to diabetic foot infections, and their diverse antimicrobial resistance profiles pose a major treatment challenge. In resource-limited settings without culture facilities, empirical treatment with antibiotics such as imipenem, piperacillin-tazobactam, vancomycin, teicoplanin, and linezolid may be initiated based on the local antibiogram. Regular updates and implementation of the local antibiogram are essential for guiding clinicians in the effective selection of antibiotics for treating diabetic foot infections. Alongside appropriate antibiotic therapy, proper ulcer care and footwear modifications remain critical for improving patient outcomes.

The present study has certain limitations that should be acknowledged. Firstly, it was conducted in a single center (tertiary care hospital) with a relatively small sample size, which may limit the generalizability of the findings to other regions; hence, a multicenter study is required to validate the findings. Secondly, the study focused only on aerobic bacterial isolates, while anaerobic bacteria were not focused on, which are important etiological agents in diabetic foot infections. Third, molecular characterization of the resistant genes was not performed, which could have provided deeper insights into the genetic mechanisms underlying the antimicrobial resistance observed in the isolates. Additionally, the study's retrospective design might have led to the loss of some patient data during retrieval.

## Conclusions

This study emphasizes a comprehensive understanding of the microbiological and clinical characteristics of patients with diabetic foot ulcers. It highlights the predominance of Gram-negative bacteria, particularly *Pseudomonas* *aeruginosa* and *Klebsiella* *pneumoniae*. The presence of multidrug-resistant bacteria highlights the increasing burden of antimicrobial resistance (AMR) and the need for judicious use of antibiotics. This retrospective study generates data regarding the common pathogens infecting diabetic foot ulcers and their antibiotic susceptibility pattern. The learning from this study will guide us in conducting prospective studies on diabetic foot ulcers more effectively.

## References

[1] Raja JM, Maturana MA, Kayali S, Khouzam A, Efeovbokhan N (2023). Diabetic foot ulcer: a comprehensive review of pathophysiology and management modalities. World J Clin Cases.

[2] Akkus G, Sert M (2022). Diabetic foot ulcers: a devastating complication of diabetes mellitus continues non-stop in spite of new medical treatment modalities. World J Diabetes.

[3] Macdonald KE, Jordan CY, Crichton E, Barnes JE, Harkin GE, Hall LM, Jones JD (2020). A retrospective analysis of the microbiology of diabetic foot infections at a Scottish tertiary hospital. BMC Infect Dis.

[4] McDermott K, Fang M, Boulton AJ, Selvin E, Hicks CW (2023). Etiology, epidemiology, and disparities in the burden of diabetic foot ulcers. Diabetes Care.

[5] Pemayun TG, Naibaho RM, Novitasari D, Amin N, Minuljo TT (2015). Risk factors for lower extremity amputation in patients with diabetic foot ulcers: a hospital-based case-control study. Diabet Foot Ankle.

[6] Darwis I, Hidayat H, Wisnu GN, Mentari S (2021). Bacteriological profile and antibiotic susceptibility pattern of diabetic foot infection in a tertiary care hospital in Lampung, Indonesia. Malays J Med Sci.

[7] Kurup R, Ansari AA (2019). A study to identify bacteriological profile and other risk factors among diabetic and non-diabetic foot ulcer patients in a Guyanese hospital setting. Diabetes Metab Syndr.

[8] Baral P, Afnan N, Zahra MA, Akter B, Prapti SR, Hossan MM, Haque FK (2024). Bacteriological analysis and antibiotic resistance in patients with diabetic foot ulcers in Dhaka. PLoS One.

[9] Turzańska K, Adesanya O, Rajagopal A, Pryce MT, Hughes DF (2023). Improving the management and treatment of diabetic foot infection: challenges and research opportunities. Int J Mol Sci.

[10] Yovera-Aldana M, Sifuentes-Hermenegildo P, Cervera-Ocaña MS, Mezones-Holguin E (2024). Association of multidrug-resistant bacteria and clinical outcomes in patients with infected diabetic foot in a Peruvian hospital: a retrospective cohort analysis. PLoS One.

[11] Ilyas F, James A, Khan S (2024). Multidrug-resistant pathogens in wound infections: a systematic review. Cureus.

[12] (2024). Antimicrobial resistance: global report on surveillance. https://www.who.int/publications/i/item/9789241564748.

[13] Bhargava B (2019). Standard Operating Procedures: Bacteriology. Second Edition. Standard Operating Procedures: Bacteriology. 2nd ed. New Delhi, India: Indian Council of Medical Research.

[14] (1996). Mackie & Mccartney Practical Medical Microbiology. Fourteenth Edition. https://cir.nii.ac.jp/crid/1971993809814398342.

[15] (2024). CLSI M100: performance standards for antimicrobial susceptibility testing. https://clsi.org/standards/products/microbiology/documents/m100.

[16] Dash RK, Mohapatra I, Singh N (2024). A five-year trend analysis of antibacterial resistance patterns among non-fermenting Gram-negative Bacilli: a retrospective study from the ICU settings of a tertiary care hospital. Cureus.

[17] Singh N, Pattnaik D, Neogi DK, Jena J, Mallick B (2016). Prevalence of ESBL in Escherichia coli isolates among ICU patients in a tertiary care hospital. J Clin Diagn Res.

[18] Verma G, Nayak SR, Jena S (2023). Prevalence of carbapenem-resistant Enterobacterales, Acinetobacter baumannii, and Pseudomonas aeruginosa in a tertiary care hospital in Eastern India: a pilot study. J Pure Appl Microbiol.

[19] Singh N, Mohanty S, Panda SS, Sahoo S, Pattnaik D, Jena J (2018). Methicillin resistant Staphylococcus aureus (MRSA) carriage among health care workers in a tertiary care hospital in Bhubaneswar. Int J Community Med Public Health.

[20] Atlaw A, Kebede HB, Abdela AA, Woldeamanuel Y (2022). Bacterial isolates from diabetic foot ulcers and their antimicrobial resistance profile from selected hospitals in Addis Ababa, Ethiopia. Front Endocrinol (Lausanne).

[21] Das A, Pendsey S, Abhyankar M, Malabade R (2020). Management of diabetic foot in an Indian clinical setup: an opinion survey. Cureus.

[22] Sudha BG, Umadevi V, Shivaram JM (2023). Diabetic foot assessment and care: barriers and facilitators in a cross-sectional study in Bangalore, India. Int J Environ Res Public Health.

[23] Pany S, Sen SK, Prasanna G, Pati S, Pal BB (2021). Spectrum of bacterial infections associated with diabetic ulcer patients. J Pure Appl Microbiol.

[24] Kale DS, Karande GS, Datkhile KD (2023). Diabetic foot ulcer in India: aetiological trends and bacterial diversity. Indian J Endocrinol Metab.

[25] Khan AA, Singh S, Singh V, Khan S (2016). Diabetic foot ulcer: a clinical study. Int Surg J.

[26] Fathima AA, Sarvotham A, Iddalagi M (2024). Study on management of diabetic foot ulcer in a rural tertiary care hospital. Indian J Vasc Endovasc Surg.

[27] Kumar U, Singh A, Singla D, Agrawal N (2023). Relationship between the duration of diabetes and severity of neuropathy in patients of peripheral neuropathic diabetic foot ulcers. Int J Res Med Sci.

[28] Boopesh S, Maharaja P, Pinto GN (2024). Clinical utility of diabetic ulcer severity score in patients with diabetic foot ulcers - a prospective study. J Med Sci Res.

[29] Li X, Qi X, Yuan G (2018). Microbiological profile and clinical characteristics of diabetic foot infection in northern China: a retrospective multicentre survey in the Beijing area. J Med Microbiol.

[30] Kim JH (2023). Investigating diabetic foot pathophysiology and amputation prevention strategies through behavioral modification. J Wound Manag Res.

[31] Smith-Strøm H, Iversen MM, Igland J (2017). Severity and duration of diabetic foot ulcer (DFU) before seeking care as predictors of healing time: a retrospective cohort study. PLoS One.

[32] Jalilian M, Sarbarzeh PA, Oubari S (2020). Factors related to severity of diabetic foot ulcer: a systematic review. Diabetes Metab Syndr Obes.

[33] Marsya V, Mahmuda IN, Lestari N, Jatmiko SW (2023). Correlations between age and hypertension on diabetic foot ulcer. Indones J Med.

[34] Zaki SM, El Karsh DS, Faden TM, Almghamsi LT, Fathaldin JO, Alhazmi OA (2024). Diabetic foot complications in Saudi Arabia: a retrospective study. Cureus.

[35] Niță O, Arhire LI, Mihalache L, Popa AD, Niță G, Gherasim A, Graur M (2023). Evaluating classification systems of diabetic foot ulcer severity: a 12-year retrospective study on factors impacting survival. Healthcare (Basel).

[36] Jawzali JI, Muhammadamin MN, Othman DY, Ikram DD (2025). Association of glycated hemoglobin levels with severity of diabetic foot ulcer and bacterial profile. J Diabetol.

[37] Zhao W, Katzmarzyk PT, Horswell R (2013). HbA1c and lower-extremity amputation risk in low-income patients with diabetes. Diabetes Care.

[38] Casadei G, Filippini M, Brognara L (2021). Glycated hemoglobin (HbA1c) as a biomarker for diabetic foot peripheral neuropathy. Diseases.

[39] Kasturi ES, Vidya GS, Hanji CV, Kumar CH (2025). Risk factors for diabetic foot ulcer among type 2 diabetes mellitus patients attending a tertiary care center - a case-control study from Central Karnataka. Indian J Community Fam Med.

[40] Shabhay A, Horumpende P, Shabhay Z (2021). Clinical profiles of diabetic foot ulcer patients undergoing major limb amputation at a tertiary care center in north-eastern Tanzania. BMC Surg.

[41] Govindaswamy S, Subbarayan E, Jaishwanth VM, Kumar SS, Santhosh S, Renji SA (2025). Bacteriological profile and antimicrobial susceptibility patterns in patients with diabetic foot infections in a tertiary care hospital in South India. J Pure Appl Microbiol.

[42] Chai W, Wang Y, Zheng H, Yue S, Liu Y, Wu Y, Li X (2021). The profile of microbiological pathogens in diabetic foot ulcers. Front Med (Lausanne).

[43] Jain SK, Barman R (2017). Bacteriological profile of diabetic foot ulcer with special reference to drug-resistant strains in a tertiary care center in North-East India. Indian J Endocrinol Metab.

[44] Alhubail A, Sewify M, Messenger G, Masoetsa R, Hussain I, Nair S, Tiss A (2020). Microbiological profile of diabetic foot ulcers in Kuwait. PLoS One.

[45] Shanmugaiah A, Pandian S, Selvam S (2020). A clinical study of commonly isolated organism and its antibiotic sensitivity in diabetic foot ulcers in Sri Manakula Vinayagar Medical College and Hospital, Pondicherry. Int Surg J.

[46] Wu M, Guo F, He X (2024). Analysis of distribution and drug susceptibility test results of pathogenic bacteria in diabetic foot ulcers. Diabetes Ther.

[47] Coşkun B, Ayhan M, Ulusoy S, Guner R (2024). Bacterial profile and antimicrobial resistance patterns of diabetic foot infections in a major research hospital of Turkey. Antibiotics (Basel).

[48] Boccella M, Santella B, Pagliano P (2021). Prevalence and antimicrobial resistance of Enterococcus species: a retrospective cohort study in Italy. Antibiotics (Basel).

[49] Costa TP, Duarte B, João AL, Coelho M, Formiga A, Pinto M, Neves J (2020). Multidrug-resistant bacteria in diabetic foot infections: experience from a Portuguese tertiary centre. Int Wound J.

[50] Woldeteklie AA, Kebede HB, Abdela AA, Woldeamanuel Y (2022). Prevalence of extended-spectrum β-lactamase and carbapenemase producers of Gram-negative bacteria, and methicillin-resistant Staphylococcus aureus in isolates from diabetic foot ulcer patients in Ethiopia. Infect Drug Resist.

[51] Madhukar M, Athavale PV, Gandham NR, Vyawahare CR, Athavale VS (2024). Commonly associated aerobic microbial pathogens and their antibiotic susceptibility profile in diabetic foot ulcers in tertiary care centre in Western Maharashtra. Indian J Med Microbiol.

